# A comprehensive candidate gene approach identifies genetic variation associated with osteosarcoma

**DOI:** 10.1186/1471-2407-11-209

**Published:** 2011-05-29

**Authors:** Lisa Mirabello, Kai Yu, Sonja I Berndt, Laurie Burdett, Zhaoming Wang, Salma Chowdhury, Kedest Teshome, Arinze Uzoka, Amy Hutchinson, Tom Grotmol, Chester Douglass, Richard B Hayes, Robert N Hoover, Sharon A Savage

**Affiliations:** 1 Division of Cancer Epidemiology and Genetics, National Cancer Institute, National Institutes of Health, Rockville, MD 20852, USA; 2 Core Genotyping Facility, National Cancer Institute, SAIC-Frederick, Inc., Gaithersburg, MD, USA; 3 Cancer Registry of Norway, PO Box 5313 Majorstuen, NO-0304 Oslo, Norway; 4 Harvard School of Dental Medicine, Boston, MA, USA; 5 Division of Epidemiology, Department of Environmental Medicine, New York University, New York, NY, USA

## Abstract

**Background:**

Osteosarcoma (OS) is a bone malignancy which occurs primarily in adolescents. Since it occurs during a period of rapid growth, genes important in bone formation and growth are plausible modifiers of risk. Genes involved in DNA repair and ribosomal function may contribute to OS pathogenesis, because they maintain the integrity of critical cellular processes. We evaluated these hypotheses in an OS association study of genes from growth/hormone, bone formation, DNA repair, and ribosomal pathways.

**Methods:**

We evaluated 4836 tag-SNPs across 255 candidate genes in 96 OS cases and 1426 controls. Logistic regression models were used to estimate the odds ratios (OR) and 95% confidence intervals (CI).

**Results:**

Twelve SNPs in growth or DNA repair genes were significantly associated with OS after Bonferroni correction. Four SNPs in the DNA repair gene *FANCM *(ORs 1.9-2.0, *P *= 0.003-0.004) and 2 SNPs downstream of the growth hormone gene *GH1 *(OR 1.6, *P *= 0.002; OR 0.5, *P *= 0.0009) were significantly associated with OS. One SNP in the region of each of the following genes was significant: *MDM2*, *MPG*, *FGF2*, *FGFR3*, *GNRH2*, and *IGF1*.

**Conclusions:**

Our results suggest that several SNPs in biologically plausible pathways are associated with OS. Larger studies are required to confirm our findings.

## Background

Osteosarcoma (OS) is the most common primary malignant bone tumor and typically occurs in adolescents and young adults (Damron *et al*, 2007; Mascarenhas L, 2006; Stiller CA, 2006). OS incidence has a bimodal age distribution; the primary peak occurs during adolescence and a second, much smaller peak is present in the elderly [1,2]. In young patients, OS incidence correlates with puberty and bone growth. The peak incidence of both OS and puberty tend to occur earlier in females. OS incidence is higher in males, who usually grow taller than females, and it typically occurs at sites of rapid bone growth (*e.g.*, the metaphyses of long bones) [1]. The incidence peak in adolescence is followed by a rapid decline and a plateau when bone growth is complete (after age 24 years) [3]. Several studies have suggested that being taller than average at diagnosis is associated with increased OS risk [4-11]. A recent meta-analysis of height at diagnosis and birth-weight as OS risk factors found that high birth-weight (OR 1.35, 95% CI 1.01-1.79, compared to average birth-weight subjects) and being taller than average were significant OS risk factors (for those ≥90^th ^percentile of height: OR 2.63, 95% CI 1.98-3.49, compared to those ≤50^th ^percentile of height) [12]. In aggregate, these data suggest that growth and development during puberty, and possibly *in utero*, contributes to OS etiology.

OS frequently occurs in several cancer predisposition syndromes, including Li-Fraumeni Syndrome [13], hereditary bilateral retinoblastoma [14], Bloom, Werner, Rothmund Thomson syndromes [15], and Diamond-Blackfan anemia [16]. It also occurs more frequently in individuals with Paget's disease [17]. However, in the majority of OS cases, there is no known predisposing factor. The data regarding the role of common germline genetic variation in OS risk are sparse. Positive or suggestive associations have been observed for SNPs in the vitamin D receptor (*VDR*; *Fok*I polymorphism) [8], tumor necrosis factor-alpha (*TNF-a*; promoter region -238 SNP) [18], insulin-like growth factor 2 receptor (*IGF2R*) [19], *Fas *[20], transforming growth factor-beta receptor 1 (*TGFBR1*) [21], and *MDM2 *[22] genes, but no associations were observed for the estrogen receptor (*ER*) [8], collagen I[alpha]1 (*COL1A1*) [8], or *TP53 *[23] genes.

Peak levels of endogenous sex hormones, growth hormones, and IGF-I levels occur during puberty which also corresponds to peak bone growth rates. It is possible that variation in genes important in bone development, growth, and puberty are modifiers of OS risk. In addition, insulin-like growth factors are known to play critical roles in carcinogenesis [24,25]. Chromosomal aneuploidy in OS cells [26,27] and the increased OS risk observed with genetic syndromes caused by mutations in DNA repair pathways [*e.g.*, *TP53 *[13], *WRN*, *BLM*, *RECQL4 *[15]] suggests that variants in DNA repair genes may be associated with OS risk. Genes in DNA repair and tumor suppressor pathways may also contribute to OS pathogenesis, because they help maintain the integrity of critical cellular processes and defects in these genes often lead to carcinogenesis. Diamond-Blackfan anemia is associated with an increased frequency of OS and mutations in ribosomal genes (*i.e.*, *RPS19*, *RPS24 *and *RPS17*) [16,28]. Thus, it is also feasible that variation in these genes may contribute to OS risk.

There are numerous genes that contribute to bone growth and puberty, and DNA repair which could contribute to OS which have not yet been evaluated. We evaluated these hypotheses in an OS association study of candidate genes from the following pathways: growth and hormone metabolism, bone formation, tumor suppressor and DNA repair, and ribosomal. We genotyped 4836 tag-SNPs across 255 candidate genes from these four pathways in 96 OS cases and 1426 cancer-free controls. This approach identified several SNPs in candidate genes from biologically plausible pathways that were associated with OS risk.

## Methods

### Study design and population

OS cases *(n *= 101) were derived from the hospital-based, prospective case-control study, the Bone Disease and Injury Study of Osteosarcoma (BDISO) [29]. Blood samples and questionnaire data on individuals were collected at orthopedic surgery departments in 10 United States medical centers between 1994 and 2000. OS patients were identified at the time of limb salvage surgery. There were no identified cases of Paget's disease of the bone in this study. Orthopedic controls from BDISO (*n *= 65) were individuals with benign tumors (26%) and other non-neoplastic conditions, such as inflammatory diseases, cysts, and trauma, excluding those with hip fracture or osteoporosis. All individuals were self-described Caucasians. Institutional review boards at each of the medical centers approved the protocol and informed consent was obtained from all study subjects.

An additional 1364 cancer-free control subjects were derived from the Prostate, Lung, Colorectal, and Ovarian (PLCO) Cancer Screening Trial. Individuals aged 55-74 years were enrolled in the screening trial between 1993 and 2001 from 10 different centers in the U.S. All subjects in this study were required to have completed a baseline questionnaire, provided a blood specimen, and consented to participate in etiologic studies of cancer and related diseases. Controls were limited to Caucasians living in the continental U.S. without a diagnosis of adenoma or cancer at baseline. The institutional review boards at the National Cancer Institute and 10 screening centers approved the study.

### Genotyping assays

DNA was isolated from blood specimens using standard methods. Genotyping was conducted on a Custom Infinium^® ^BeadChip (iSelect)™ from Illumina, Inc. The iSelect panel was created by investigators in the Division of Cancer Epidemiology and Genetics, National Cancer Institute (NCI) to target genetic variation in genes potentially important in carcinogenesis and cancer risk. Tag SNPs were identified from the HapMap CEU population assuming a r^2 ^threshold of 0.80 using the Tagzilla module of the GLU software package (http://code.google.com/p/glu-genetics/) across 255 candidate genes, including the region 20 kb upstream and 10 kb downstream from the gene. Additional potentially functional SNPs were forced into the tag-SNP selection for select genes. In this study, a total of 6050 tag-SNPs were genotyped.

The concordance rates between 10 duplicate BDISO and PLCO samples on the iSelect panel were 99.5% and 99.9%, respectively. SNPs were excluded if they had less than a 90% genotyping rate in either study population, were non-variable or had a minor allele frequency (MAF) <1%, or if they failed the Hardy-Weinberg equilibrium test or genotyping validation. Individuals were excluded if they had too much missing genotype data (>10% missing genotypes). A total of 4836 tag-SNPs met these quality control criteria and were included for analysis.

After selecting approximately 500 genetically matched controls, based on 28 k iSelect SNPs, from our 1426 controls and comparing these results to the results using all 1426 controls, we determined that there was no significant difference in the results; and thus, we used all 1426 controls in our analyses to maximize our ability to detect associations with rare alleles. A principal component analysis was performed using a set of 3,843 structure inference SNPs selected from the iSelect BeadChip (27,905 SNPs) to evaluate population substructure among the BDISO individuals and the PLCO controls. There was no evidence of significant population stratification. However, 5 cases, 2 orthopedic controls and 1 PLCO control were considered genetic outliers and excluded from the genotyping analyses for a final sample size of: 96 cases, 63 orthopedic controls, and 1363 PLCO controls.

### Statistical analyses

Logistic regression models were used to estimate the odds ratio (OR) and 95% confidence intervals (CI) for the strength of the association between OS risk independently for each SNP, adjusting for gender. To deal with rare variants, the most appropriate method was chosen in the sequence of logistic regression on the additive trend model, logistic regression on the dominant model, or the Fisher's Exact Test. The most common allele or the homozygote of the common allele was used as the referent category for the additive or dominant model, respectively. Bonferroni corrections (*P*_adj_) were conducted by gene (for all SNPs in a gene) for correction of multiple statistical tests.

Genes were categorized into biological pathways using the Kegg pathway database (http://www.genome.jp/kegg/pathway.html) or by function based on literature review. We conducted gene-level and pathway-level analyses based on Yu *et al *[30]. The gene-level analysis is a global test for the association between the outcome and a subset of SNPs within a given gene or region. The pathway-level analysis is a global test for the association between the outcome and any subset of genes within a given pathway. *P*-values for these analysis were estimated with 20,000 permutation steps according to the algorithm given in Yu *et al *[30].

Statistical analyses were performed using SAS software, version 9.1 (SAS Institute, Cary, NC), R language, and PLINK software, version 1.06 (http://pngu.mgh.harvard.edu/purcell/plink/). We evaluated the correlation between SNPs [linkage disequilibrium (LD)] across specific gene regions with Haploview version 4.1 [31].

## Results

The characteristics of the study participants are shown in Table 1. The median age of the 96 OS cases was 20.3 years (age range 8 to 80.5). The median age of the 63 orthopedic controls from the BDISO was 18.5 years (age range 7.2-68.5), and the PLCO controls were older with a median age of 62 years (age range 55-75). All participants were self-identified Caucasians and from the continental United States. Additional file 1, Table S1 shows the genes analyzed in each pathway, the number of SNPs and most significant SNP in each gene, and the gene- and pathway-level *P *values. There were 161 genes (2835 SNPs) in the DNA repair pathway, 62 genes (1448 SNPs) in the growth/hormone metabolism pathway, 28 genes (534 SNPs) in the bone formation pathway, and 4 genes (19 SNPs) in the ribosomal pathway. We took three approaches to the analyses by evaluating associations with OS at the individual SNP level, gene level, and pathway level. We used the conservative Bonferroni correction to correct for multiple statistical tests.

**Table 1:** Characteristics of cases and controls

	mean age (SD)	*n *(%) male	*n *(%) female	total *n*
OS Cases	26.6 (16.5)	54 (56.3)	42 (43.7)	96
All Controls	60.9 (9.9)	904 (63.4)	522 (36.6)	1426
Orthopedic Controls	24.7 (15.1)	34 (54.0)	29 (46.0)	63
PLCO Controls	62.6 (5.2)	870 (63.8)	493 (36.2)	1363

*n *= number of individuals;

SD = standard deviation;

PLCO = Prostate, lung, colon, ovarian cancer cohort.

### Individual SNPs associated with osteosarcoma

**Figure 1:**
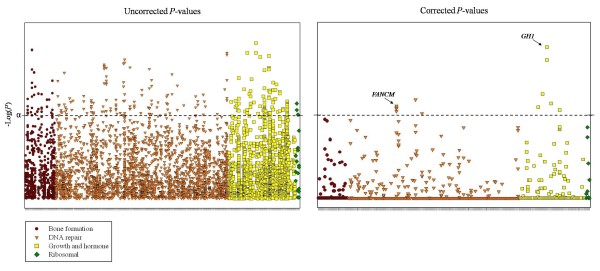
**Plot of *P *values for each SNP by functional pathway before and after Bonferroni correction for the number of SNPs per gene**. Inset shows the pathway designations. 4836 SNPs in 255 candidate genes are shown. α = 0.05; dashed line represents an extension of α.

Of the 4836 SNPs, 241 (expected is 241.8) were statistically significant (*P *< 0.05) before correction for multiple tests (Additional file 2, Table S2). A SNP downstream of *GH1 *(growth hormone 1) was the most significantly associated with OS (*P*_trend _= 0.0009). Twelve SNPs, all in genes from the DNA repair or growth and hormone pathways, were significantly associated with OS after Bonferroni correction (*P*_adj _< 0.05) for multiple tests by gene (for all SNPs in a gene; Table 2 and Figure 1). There were 4 SNPs in the DNA repair gene *FANCM *(Fanconi anemia, complementation group M) and 2 SNPs in the growth and hormone gene *GH1 *that were significantly associated with OS after correction for multiple tests. The 4 significant SNPs in *FANCM *were not highly correlated using our control data (D' = 0.93-1.0 and r^2 ^= 0.01-0.37) and HapMap CEU population data (Additional file 3, Figure S1). There was high LD among the 2 significant SNPs downstream of *GH1 *with our control data (D' = 0.96 and r^2 ^= 0.91) and HapMap CEU population data (Additional file 3, Figure S2). In Figure 1, SNPs in *GH1 *clearly appear more significant after correction than SNPs in any other genes. Five SNPs in the upstream or downstream region of candidate genes were significant after correction: downstream of *MDM2 *[Mdm2 p53 binding protein homolog (mouse)], and *FGF2 *[fibroblast growth factor 2 (basic)], and upstream of *MPG *(N-methylpurine-DNA glycosylase), *FGFR3 *(fibroblast growth factor receptor 3), and *GNRH2 *(gonadotropin-releasing hormone 2). A SNP in intron 2 (IVS2+10605) of *IGF1 *[insulin-like growth factor 1 (somatomedin C)] was significantly associated with a decreased risk of OS after correction.

**Table 2:** Significant SNPs after Bonferroni correction by gene (*P*_adj_)

Pathway	Gene	SNP	Genomic position		Minor Allele	MAF (%) Controls	MAF (%) Cases	**OR**^**†**^	95% CI	*P*	***P***_**adj**_	**Model or Test**^**§**^	Gene *P*
DNA repair	*FANCM*	rs1367580	Chr14: 44714339	Ex14+316 (V878L)	T	10.4	16.7	1.97	(1.26-3.08)	0.0031	0.034	Dominant	0.019
		rs11845507	Chr14: 44721715	IVS16-1012	A	10.5	16.7	1.96	(1.25-3.06)	0.0033	0.037	Dominant	
		rs4900664	Chr14: 44732264	IVS20-2861	T	9.3	15.3	2.00	(1.25-3.17)	0.0035	0.038	Dominant	
		rs7141145	Chr14: 44733578	IVS20-1547	A	10.3	16.5	1.95	(1.24-3.07)	0.0039	0.043	Dominant	
	*MDM2*	rs1690916	Chr12: 67521673	downstream, no gene	A	42.5	31.3	0.62	(0.45-0.85)	0.0029	0.026	Additive	0.016
	*MPG*	rs216614	Chr16: 60334	upstream, in *RHBDF1*	A	0.7	3.1	4.80	--	0.0036	0.047	Fisher's	0.027

Growth and hormone	*FGF2*	rs11737764	Chr4: 124046230	downstream, in *NUDT6*	T	8.1	14.1	2.12	(1.33-3.39)	0.0016	0.036	Dominant	0.020
	*FGFR3*	rs6599400	Chr4: 1754823	upstream, no gene	A	33.0	42.6	1.51	(1.12-2.03)	0.0069	0.021	Additive	0.017
	*GH1*	rs11079515	Chr17: 59359377	downstream, no gene	G	37.9	49.5	1.61	(1.20-2.16)	0.0016	0.005	Additive	0.002
		rs7921	Chr17: 59359991	downstream, in *CD79B*	A	27.2	16.1	0.52	(0.35-0.76)	0.0009	0.003	Additive	
	*GNRH2*	rs3761243	Chr20: 2971022	upstream, no gene	C	29.7	40.5	1.60	(1.19-2.16)	0.0020	0.031	Additive	0.021
	*IGF1*	rs7956547	Chr12: 101382946	IVS2+10605	C	26.3	15.8	0.53	(0.36-0.79)	0.0019	0.040	Additive	0.021

MAF = minor allele frequency;

^† ^Odds ratios were estimated using logistic regression models with the most common allele or genotype as the referent, adjusted for gender;

^§ ^Results are shown for the model or test chosen to deal with rare variants in the sequence of logistic regression on the additive trend model, logistic regression on the dominant model, or the Fisher's Exact Test.

### Genes and pathways associated with osteosarcoma

We evaluated 255 candidate genes from four functional pathways (Additional file 1, Table S1). Fourteen genes were significantly associated with OS (Gene *P*-values <0.05; Additional file 1, Table S1 and Additional file 2, Table S2). The most significantly related genes were *GH1 *(Gene *P *= 0.002), *MDM2 *(Gene *P *= 0.016), *FGFR3 *(Gene *P *= 0.017), and *FANCM *(Gene *P *= 0.019). However, if we correct for multiple tests (255 genes), none remain significant. None of the four pathways were significantly associated with OS (Additional file 1, Table S1).

## Discussion

The biology of OS pathogenesis is complex and there are limited data on risk factors in the more common sporadic form of OS. Epidemiologic studies of OS suggest that growth and development play a role in etiology [2,32,33]. It occurs primarily in adolescents during puberty [1] when bone growth is rapid and endogenous sex hormones and growth hormones are at their highest, so variation in a gene involved in regulating sex hormones is biologically plausible. Rapidly growing tissue, such as bone during puberty, is known to be highly susceptible to carcinogenesis, possibly due to rapidly proliferating osteogenic cells being more vulnerable to DNA repair errors [4,34]. In addition, chromosomal aneuploidy is extensive in somatic OS cells, which suggests the presence of chromosomal instability[26,27]. The increased frequency of OS in genetic predisposition syndromes [13,15] characterized by mutations in DNA repair pathways suggests that variants in genes involved in DNA repair are also reasonable candidates.

We evaluated the association between OS and 255 candidate genes, including 4836 SNPs, from four functional pathways (growth and hormone metabolism, bone formation, DNA repair, and ribosomal). We used 3 approaches to comprehensively evaluate these biologically plausible pathways: analyses were performed at the individual SNP level, gene level and pathway level with conservative statistical corrections for multiple testing. While no genes or pathways were significantly associated with OS after correction for multiple tests, the SNP based approach identified some potentially important candidates. A total of twelve SNPs in genes from the growth and hormone metabolism, and DNA repair pathways were significantly associated with OS risk after correction for multiple tests. Two genes had multiple significant SNPs associated with risk, *FANCM *and *GH1*, after correction for multiple tests.

*FANCM *contained 4 SNPs significantly associated with a similar 2-fold increased risk of OS using a dominant inheritance model, the most in any gene studied. These SNPs were not correlated in our controls. One SNP is located in exon 14 and the minor or risk allele results in a nonsynonymous change from valine to leucine (Ex14+316, Val878Leu). The minor allele of this SNP is the ancestral allele and is highly conserved among other mammalian species. The three other SNPs in *FANCM *were intronic. FANCM has DNA-dependent ATPase activity, promotes the dissociation of DNA triplexes, and with other Fanconi anemia-associated proteins, may repair DNA at stalled replication forks [35,36]. DNA repair must be accurate to preserve genome stability for long-term cellular viability; genetic instability is characteristic of cancer cells, and may be due, at least in part, to mutations or variation in genes that function to ensure DNA integrity [37].

Two significant SNPs were located downstream of *GH1*. They appear to be highly correlated in our controls, although one SNP was associated with an increased risk of OS and the other was protective. The variant in *IGF1 *(rs7956547, IVS2+10605) associated with a decreased risk of OS was another interesting candidate involved in growth. The insulin-like growth factor signaling system is important in the formation and homeostasis of bone, and differential expression of IGF1 has been observed in osteosarcomas [38-40]. IGFI expression is stimulated by growth hormone, and OS incidence peaks during puberty with the release of growth hormone. OS cells have been shown to be IGF1-dependent for growth, and inhibiting growth hormone release in mice decreased IGF1 serum levels and inhibited tumor growth [41-43]. In addition, animal model data from dogs, which develop OS similar to human patients (similar sites, histology and treatment response) and large breeds have a 185-fold increased risk of OS compared with small dog breeds [44,45], suggest that a SNP in *IGF1 *is a main determinant of small size in dogs and is virtually absent in giant breeds [46]. The data suggest that *GH1 *and *IGF1 *may play a role in the etiology of OS.

We also specifically evaluated the genes found to be associated with OS in other studies. We previously identified a SNP in *IGF2R *(rs998075; Ex16+88G >A) to be significantly associated with OS risk in study of variation in genes critical in growth regulation [19]. This SNP was also included in our dataset and the significant association replicated in an analysis limited to our BDISO cases and controls (*P *= 0.01), as expected because it is the same study population. However, the association did not replicate with the addition of our 1363 PLCO controls (*P *= 0.12), which suggests that the original study may have been limited by the number of controls, or possibly these older PLCO controls have a different ethnic mix within whites related to this polymorphism. Others have found significant associations between OS and polymorphisms in *VDR *[8], *TGFBR1 *[21], and *MDM2 *[22], which were also included in our dataset. We found no significant associations with individual SNPs within *VDR *or *TGFBR1 *before or after correction for multiple tests, although our dataset did not include the *VDR Fok*I polymorphism [8] or TGFBR1*6A variant [21], or at the gene level. One SNP downstream of the DNA repair gene *MDM2*, rs1690916, was significantly associated with a decreased risk of OS after correction (OR 0.62, 95% CI 0.45-0.85, *P*_adj _= 0.026), and 3 intronic SNPs were significantly associated with an increased risk of OS before correction (ORs 1.6-1.8, *P *= 0.008-0.02). The previously identified [22]*MDM2 *T309G (rs2279744) polymorphism was marginally non-significantly associated with an increased risk of OS (OR 1.31, 95% CI 0.97-1.77, *P *= 0.07). At the gene level, *MDM2 *was found to be significantly associated with OS (Gene *P *= 0.016), but not after correction for multiple tests. As others have shown [8,23], the current study did not confirm associations between polymorphisms in *ESR1/2 *(including previously analyzed PvuII and XbaI polymorphisms), *COL1A1*, or *TP53 *and OS after correction, or at the gene level.

We also investigated many of the genes with germline mutations in cancer predisposition syndromes associated with an increased frequency of OS [15], including the DNA repair genes *TP53 *(mutated in Li-Fraumeni Syndrome), *WRN *(mutated in Werner syndrome), *BLM *(mutated in Bloom syndrome), *RECQL4 *(mutated in Rothmund-Thomson Syndrome), and the ribosomal genes *RPS19 *and *RPS24 *(mutated in Diamond-Blackfan anemia) [28,47,48]. OS also occurs more commonly in older individuals with Paget's disease[17]. Two genes involved in bone formation, *SQSTM1 *and *TNFRSF11A*, have been shown to cause Paget's disease [49]. We found that no common SNPs within these genes that predispose to cancer predisposition syndromes or Paget's disease were significantly associated with OS after correction for multiple tests.

A limitation of the current study was the small number of cases; however, the inclusion of 14 controls per case improved the statistical power to detect SNPs with strong effects. For the additive model and our 96 cases and 1426 controls, we had greater than 80% statistical power to detect an OR of 1.82 for MAFs of 0.1 and an OR of 2.15 for MAFs of 0.05 (with a baseline population risk of 0.0000001 and type 1 error of 0.05). Another potential limitation of our study was the use of controls from PLCO that were older than the BDISO case and control population. However, the PLCO controls had no history of any cancer, including osteosarcoma, they were limited to Caucasians from the continental US, as were the BDISO cases and controls, and we found no evidence of population stratification in between groups.

Strengths of the current study include the detailed genotyping of biologically plausible pathways which give a higher a priori likelihood. We used three statistical approaches to comprehensively evaluate associations with OS (at the SNP level, gene level, and pathway level). In addition, we used a stringent Bonferroni correction and conservatively interpreted our results to reduce the probability of a Type 1 error.

## Conclusions

We have conducted the largest study of genetic variation in candidate genes associated with OS to date. We identified the presence of genetic variants in candidate genes from biologically plausible pathways important in growth and hormone metabolism, and DNA repair associated with OS, even with conservative statistical corrections. The strongest candidates are *FANCM*, *GH1 *and *IGF1*. The potential functional implications of the variation in these genes are currently unknown. However, since OS occurs during a period of rapid bone growth, genes important in growth, puberty, and DNA repair are biologically plausible contributors to OS pathogenesis because of their function in critical cellular processes. Larger studies of common genetic variation in OS and functional studies of the SNPs identified here are required to confirm the significance of our findings.

## Competing interests

The authors declare that they have no competing interests.

## Authors' contributions

LM helped with the study design, performed the statistical analyses, and drafted the manuscript. KY conducted the global gene and pathway analyses. SIB and RBH designed the genotyping component of the Prostate, Lung, Colorectal, and Ovarian Cancer Screening Trial and provided genotype data for these participants. LB, ZW, SC, KT, AU, and AH conducted all of the genotyping assays. TG participated in manuscript preparation. CD and RNH designed the Bone Disease and Injury Study of Osteosarcoma. SAS designed the study, provided input to the analysis strategy, and participated in manuscript preparation. All authors read and approved the final manuscript.

## Pre-publication history

The pre-publication history for this paper can be accessed here:

http://www.biomedcentral.com/1471-2407/11/209/prepub

## Supplementary Material

Additional file 1 **Table S1**. SNPs from 255 candidate genes by functional pathway. This table shows the genes analyzed in each pathway, the number of SNPs in each gene, the *P *value for the most significant SNP in each gene, and the gene- and pathway-level *P *values. Click here for file

Additional file 2 **Table S2**. Significant SNPs (*P *< 0.05) in each pathway associated with osteosarcoma before correction for multiple tests. This table lists the statistics (minor allele, MAF, OR, and *P *values) for each of the 241 SNPs significantly (*P *< 0.05) associated with osteosarcoma before correction for multiple tests by gene and pathway. Click here for file

Additional file 3 **Supplementary Figures S1 and S2.** Figure S1. Linkage disequilibrium across *FANCM *using the HapMap Caucasian (CEU) population data (A), and our control data (B) determined using Haploview. This figure illustrates the linkage disequilibrium across *FANCM *and highlights the significant SNPs associated with osteosarcoma after correction for multiple tests. Figure S2. Linkage disequilibrium across *GH1 *using the HapMap Caucasian (CEU) population data (A), and our control data (B) determined using Haploview. This figure illustrates the linkage disequilibrium across *GH1 *and highlights the significant SNPs associated with osteosarcoma after correction for multiple tests Click here for file
