# Supplementary material for: A comprehensive candidate gene approach identifies genetic variation associated with osteosarcoma
Source: BMC Cancer. 2011 May 29;11:209. doi: 10.1186/1471-2407-11-209 (PMC3138419; doi:10.1186/1471-2407-11-209)
Supplement: Additional file 3 — Supplementary Figures S1 and S2. Figure S1. Linkage disequilibrium across FANCM using the HapMap Caucasian (CEU) population data (A), and our control data (B) determined using Haploview. This figure illustrates the linkage disequilibrium across FANCM and highlights the significant SNPs associated with osteosarcoma after correction for multiple tests. Figure S2. Linkage disequilibrium across GH1 using the HapMap Caucasian (CEU) population data (A), and our control data (B) determined using Haploview. This figure illustrates the linkage disequilibrium across GH1 and highlights the significant SNPs associated with osteosarcoma after correction for multiple tests [file 1471-2407-11-209-S3.DOC]

**Supplemental Figures S1 and S2**

**Figure S1.** Linkage disequilibrium across *FANCM* using the HapMap Caucasian (CEU) population data (A), and our control data (B) determined using Haploview. SNPs significantly associated with osteosarcoma after correction for multiple tests are underlined. This region represents 20kb upstream and 10kb downstream from chromosome 14 nucleotides 44,674,900 to 44,739,837.

**A)**


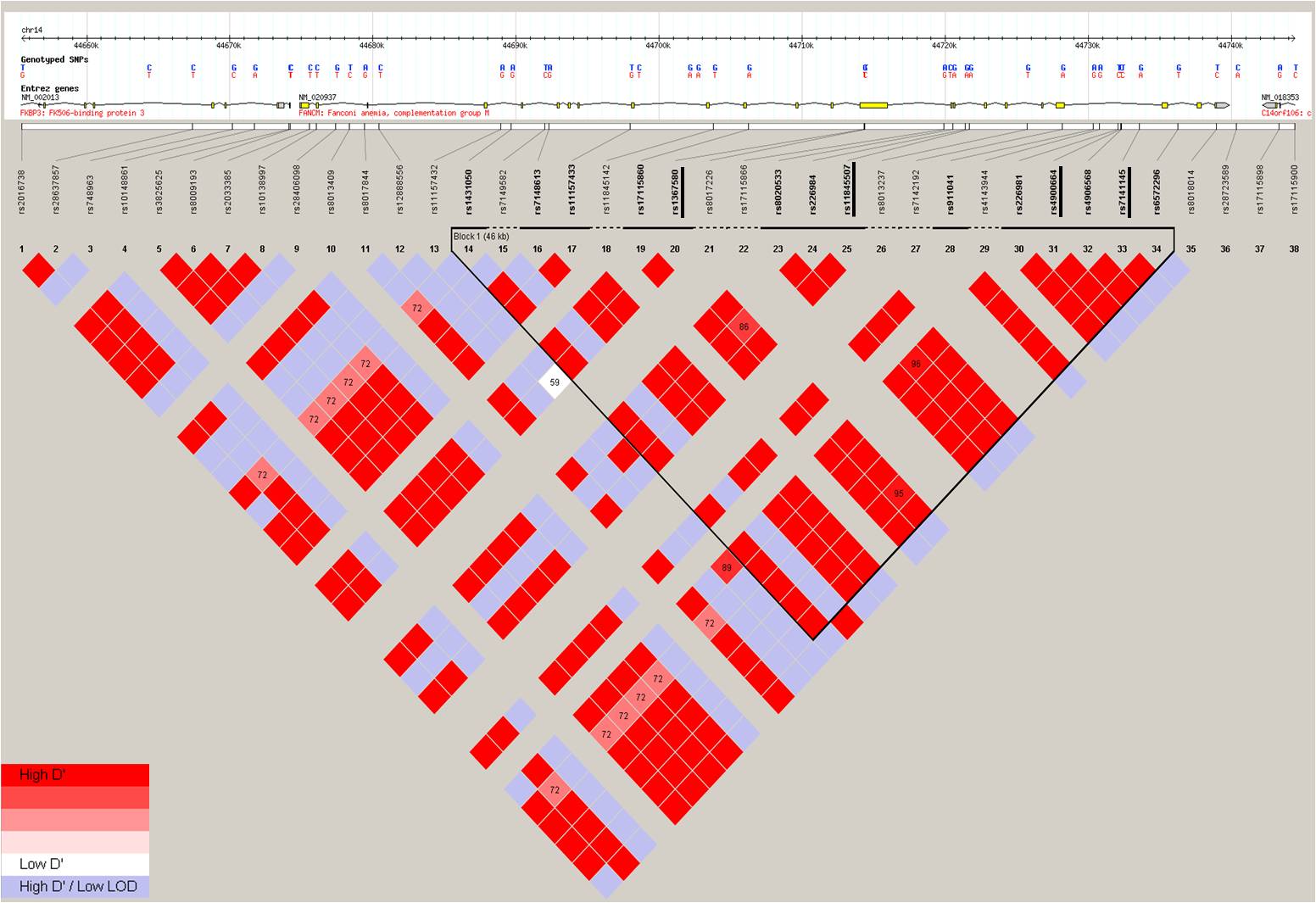


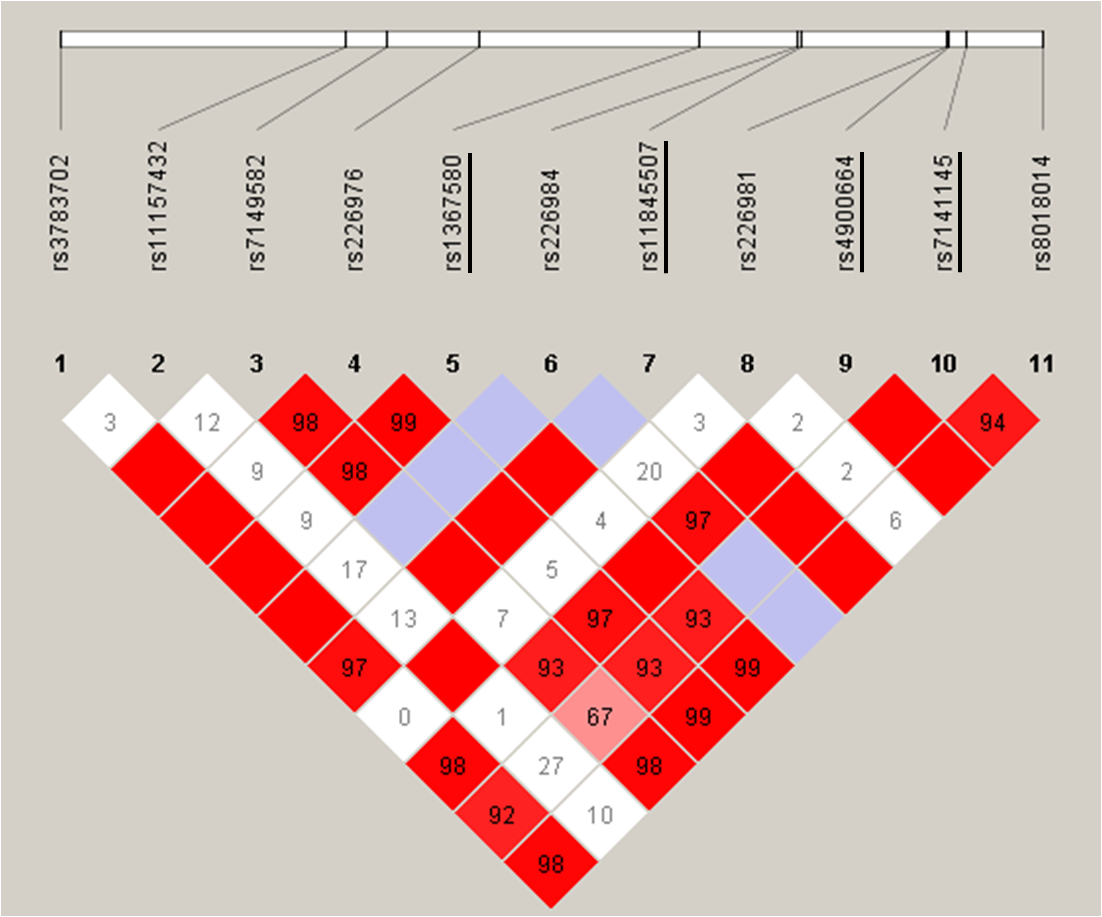


**B)**

**Figure S2.** Linkage disequilibrium across *GH1* using the HapMap Caucasian (CEU) population data (A), and our control data (B) determined using Haploview. SNPs significantly associated with osteosarcoma after correction for multiple tests are underlined. Only 1 of the 2 SNPs in *GH1* significantly associated with osteosarcoma was included in HapMap. This region represents 20kb upstream and 10kb downstream from chromosome 17 nucleotides 59,348,296 to 59,349,930. The HapMap plot, under Entrez genes, does not list the isoforms for *GH1*.

**A)**


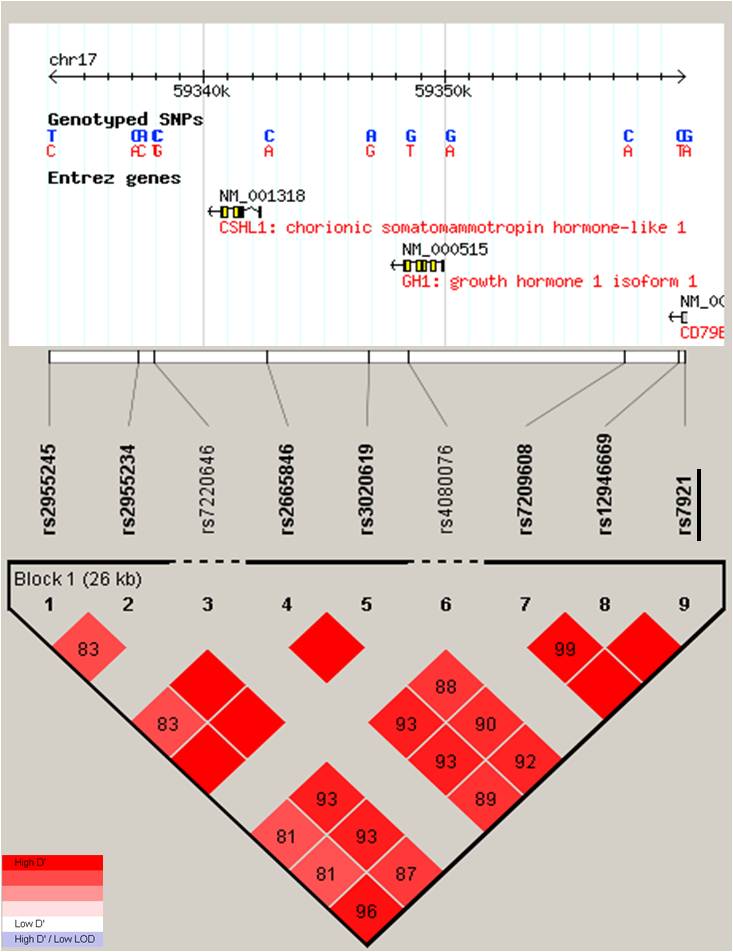


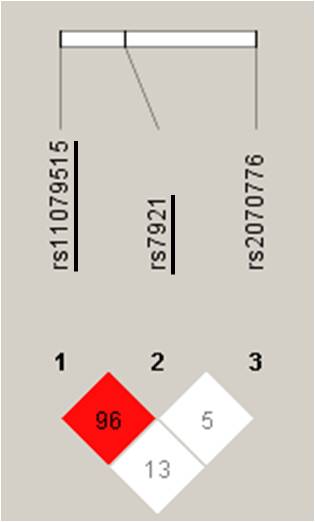


**B)**
